# Challenges With Buprenorphine Initiation in the Fentanyl Era—Adapting Effectively to the Moment

**DOI:** 10.1001/jamanetworkopen.2025.52140

**Published:** 2026-01-02

**Authors:** William M. Garneau, Eric C. Strain, Michael I. Fingerhood

**Affiliations:** Department of Medicine, Division of Hospital Medicine, Johns Hopkins University School of Medicine, Baltimore, Maryland; Behavioral Pharmacology Research Unit, Department of Psychiatry and Behavioral Sciences, Johns Hopkins University School of Medicine, Baltimore, Maryland; Department of Medicine, Division of Addiction Medicine, Johns Hopkins University School of Medicine, Baltimore, Maryland

Current best practice for management of opioid use disorder (OUD) includes provision of medication with full (eg, methadone) or partial (eg, buprenorphine) activity at the μ opioid receptors. Buprenorphine is extensively used to treat OUD, and has long been noted to have the capacity to precipitate opioid withdrawal with initial dosing.^1^ In this study, Kawasaki et al^2^ share data from a nationwide survey of buprenorphine prescribers reporting challenges with buprenorphine-related withdrawal in the current era of nonpharmaceutical fentanyl (NPF) use.^2^ These findings are timely and illustrate the complexity of contemporary treatment of OUD.

The reported incidence of buprenorphine initiation–related precipitated withdrawal (PW) in the era of fentanyl has varied widely. A nationwide clinical trial of emergency department–initiated buprenorphine reported a less than 1% incidence of PW, while other recent accounts have included rates of more than 10%.^3,4^ In this study, Kawasaki et al^2^ report the findings of a 2023 to 2024 nationally representative survey of 396 US buprenorphine prescribers. The study population included physicians and advanced practice clinicians who initiated at least 10 patients with OUD onto buprenorphine during the prior year and at least 1 in the past 90 days. The survey included 96 questions covering clinician demographics, patient characteristics, and clinical practice and setting. The primary outcome was the percentage of respondents reporting problems initiating buprenorphine in the context of NPF, either due to precipitated or prolonged withdrawal.

Almost three-quarters of respondents (72.0%) reported experiencing challenges in starting buprenorphine in the past year. Two-thirds (67.3%) of clinicians reported altering their standard initiation protocols, and 61.4% reported 1 or more episodes of PW. Concerningly, almost 10% of respondents reported that up to 50% of their patients could not successfully initiate buprenorphine and required their treatment be changed to methadone. Clinicians who were initiating patients in noninpatient settings were especially affected. This is notable, as one feature of noninpatient initiation is the ability to start with lower and slower dosing (eg, one-quarter of a 2 mg filmstrip [ie, 0.5 mg] at a time).

While it is unlikely the US will go back to seeing people with lower levels of opioid physical dependence, buprenorphine is, and remains, a critically important part of the solution. Rather than avoiding this lifesaving option, we must acknowledge the risk of PW is increasing in the setting of more potent drugs, such as NPFs, and take action to preserve trust in this treatment, both among clinicians, and more importantly, among persons with OUD. Reports of being allergic to buprenorphine, or patients who say buprenorphine does not work for them reflect lived experience. In some cases, methadone may be appropriate; however, the flexibility of buprenorphine, with its safety profile and ease of patient access, make it invaluable.

Exploratory work is being done to respond to this evolving landscape of illicit drug use: low (or micro) and macro initial buprenorphine dose approaches are being investigated using sublingual, transdermal, and long-acting injectable routes.^5–7^ In the emergency medical services setting, macrodosing buprenorphine for individuals who have received naloxone in the field is an area of important work and has an existing gap in evidence. Closer supervision on the day of initiation may be needed. Clinical data are being collected and can help to inform optimal new dose initiation procedures; however, larger pragmatic trials are urgently needed to guide clinicians on how to safely and reliably start this lifesaving medication.

Importantly, Kawasaki et al^2^ have provided data that begin to address the scope of the problem, and they have shown that the issue of PW is no longer a rare phenomenon. These findings suggest that clinicians practicing in the field of addiction commonly encounter difficulty initiating buprenorphine in patients who have prior exposure to NPF, and clinicians are modifying their initiation on an ad hoc basis responding to this. While clinicians are seeking ways to address this issue, controlled studies are needed to inform practice.

We know that buprenorphine saves lives. Our next step is to develop the evidence base for buprenorphine initiation in the fentanyl era to safely and reliably support patients making the first steps in recovery. Kawasaki and colleagues^2^ have provided important data on the current experience when using this medication. While there may be challenges in starting some patients with buprenorphine, it is important to keep in mind that most patients are successfully started on buprenorphine, and that the challenges in starting it seen in some patients are greatly outweighed by the benefits that can accrue through use of buprenorphine as an aid in the road to recovery for persons with OUD.
